# Cervical Necrotizing Fasciitis: A Surgical Outcome Analysis

**DOI:** 10.7759/cureus.44678

**Published:** 2023-09-04

**Authors:** Shaila Sidam, Abhinav Bhagat, Aparna Chavan, Anjan K Sahoo

**Affiliations:** 1 Otolaryngology - Head and Neck Surgery, All India Institute of Medical Sciences, Bhopal, Bhopal, IND; 2 Radiodiagnosis, All India Institute of Medical Sciences, Bhopal, Bhopal, IND; 3 Department of Otorhinolaryngology, RKDF Medical College Hospital & Research Center, Bhopal, IND

**Keywords:** broad-spectrum antibiotics, mortality, neck exploration, debridement, cervical necrotizing fasciitis

## Abstract

Cervical necrotizing fasciitis is an immensely progressive, difficult-to-diagnose soft tissue infection of the fascial planes, skin, and subcutaneous tissue. It has marked morbidity and mortality. In this case report, we analyzed the risk factors, laboratory indices, and treatment modalities that affect the outcome of this fatal disease. This is a retrospective case series of cases admitted within a short span of six months between January and June 23. The cases were followed up monthly for three months, and the diagnosis was made on a clinical, pathological, radiological, and histopathological basis. All the cases were managed with neck exploration and aggressive surgical debridement in an emergency department, dressing of the wound with hydrogen peroxide and betadine twice daily, triple broad-spectrum antibiotic therapy for polymicrobial infection, and tight glycemic control. There were no complications, and all the patients survived. We report our cases of cervical necrotizing fasciitis that had similar presentations but varied outcomes. Here, we would like to advocate the importance of immediate management in the form of neck exploration and debridement at the earliest after the diagnosis has been established. Hyperglycemia should be brought under control, and daily aseptic dressing with removal of the slough and source of infection would greatly affect the outcome of this deadly disease.

## Introduction

Cervical necrotizing fasciitis is an uncommon fulminating progressive infection affecting the soft tissue of the fascial planes of the head and neck. The incidence in the head and neck is 1-10% [1]. It is mostly seen in the abdomen, groin, and perineum and the extremities. The infection affects patients with compromised immunity, and the most frequent cause of infection is odontogenic or pharyngeal [2]. It is caused by mixed flora of aerobes and anaerobes. The high fatality is due to close proximity to vital anatomical structures, leading to early systemic toxicity, such as airway compromise, sepsis, and mediastinitis [3].

Therefore, prompt early diagnosis with interventions in the form of neck exploration and debridement until healthy tissues are seen, securing the airway, culture-directed broad-spectrum antibiotics, strict glycemic control, and frequent aseptic dressing should be done.

Here, we attempted to analyze case records with diagnosis of cervical necrotizing fasciitis to determine the factors affecting the outcome of this lethal disease.

## Case presentation

Materials and methods

This is a case report of cases of cervical necrotizing fasciitis, with inclusion criteria of tender neck swelling with skin discoloration or intraoperative finding of necrosis during neck exploration. Only biopsy-proven cases were included. All other cases of abscess were excluded. All cases underwent emergency surgery and radiological examination, except one (case 2). Clinical and demographic details were noted, like comorbidity, laboratory indices, radiology, culture sensitivity, histopathology, antibiotics administered, duration of hospital stay, complications, outcome, and survival.

Only four cases, one male and three female cases, fulfilled the inclusion criteria, which may be inadequate to draw any conclusion. Among the four cases, only three had diabetes, and case 3 was non-diabetic and not immunocompromised. On the basis of the histopathological report and clinical presentation of skin discoloration and intraoperative finding of slough and necrotic tissue, the cases were diagnosed. All the cases underwent emergency neck exploration and debridement, with regular wound dressing with hydrogen peroxide and betadine twice daily, because hydrogen peroxide exhibits antimicrobial properties due to free hydroxyl radicals and povidone iodine has antiseptic microbicidal action. All the cases were started on empirical broad-spectrum antibiotics consisting of amoxicillin-clavulanate, metronidazole, and amikacin. Then, as per the culture sensitivity report, they were shifted to culture-directed antibiotics for seven to 10 days in the case of non-diabetics and for 10 days or more in the cases of diabetics, and oral antibiotics were given for seven days on discharge. As per the need, only one case underwent ancillary procedure, such as split skin grafting.

Results 

Among the four cases, three cases were above 40 years, one male and two female cases, and another female was less than 40 years. Three cases were of uncontrolled diabetes mellitus, and all cases had anemia. All cases had an odontogenic source of infection based on the patients' medical history, and all cases underwent tooth extraction. The culture report of pus sent from the neck of case 1 was positive for *Acinetobacter baumannii* and *Pseudomonas aeruginosa*. All presented with tender neck swelling with skin discoloration, sometimes crepitus or surgical emphysema due to thrombosis of blood vessels, which can cause gas gangrene. In all four cases, the submandibular, submental spaces were involved with unilateral neck swelling in two cases and bilateral neck in two, which extended to the upper chest with the involvement of other spaces, such as carotid and parapharyngeal spaces.

Laboratory indices demonstrated that all cases had anemia, hyponatremia, hypoalbuminemia, neutrophilia, and leukocytosis, which is an increase in white blood cells (WBCs) in the blood (normal range 4-11): case 1 had 20.78, case 2 had 22.40, case 3 had 15.45, and case 4 had 15. All four cases had raised C-reactive protein (CRP) inflammatory markers. Hyperglycemia with elevated HbA1C levels was noted in three cases. The pus culture was sterile in three cases, and in the third, it was positive for *Acinetobacter baumannii *complex and *Pseudomonas aeruginosa*. The histopathology in all cases, as depicted in Figure 2 for case 1, Figure 3 for case 2, Figure 5 for case 3, and Figure 7 for case 4, was consistent with cervical necrotizing fasciitis. In two cases, the duration of hospital stay was 20 days, while in the others, it was one month and 20 days. All the cases survived with no complications. The results of all the cases are depicted in Table 1.

**Table 1:** Details of factors affecting survival in cervical necrotizing fasciitis HbA1c, glycated hemoglobin; N/A, not applicable

S. No.	Parameters	Case 1	Case 2	Case 3	Case 4
1	Age/sex	48 years/male	41 years/female	34 years/female	48 years/female
2	Etiology	Dental	Dental	Dental	Dental
3	Comorbidity	Diabetic	Diabetic	Non-diabetic	Diabetic
4	Leukocytosis	Yes	Yes	Yes	Yes
5	C-reactive protein	Elevated	Elevated	Elevated	Elevated
6	Neutrophilia	Yes	Yes	Yes	Yes
7	Anemia	Yes	Yes	Yes	Yes
8	Hyperglycemia	Yes	Yes	No	Yes
9	HbA1c	Elevated	Elevated	Normal	Elevated
10	Hypoalbuminemia	Yes	Yes	Yes	Yes
11	Bacteriology	*Acinetobacter baumannii *complex and *Pseudomonas aeruginosa*	Sterile	Sterile	Sterile
12	Histopathology	Consistent with cervical necrotizing fasciitis	Consistent with cervical necrotizing fasciitis	Consistent with cervical necrotizing fasciitis	Consistent with cervical necrotizing fasciitis
13	Radiology	CT scan		CT scan	CT scan
14	Added procedure	N/A	Split skin grafting	N/A	N/A
15	Complication	No	No	No	No
16	Duration of stay	One month and 20 days	20 days	20 days	20 days
17	Outcome	Survived	Survived	Survived	Survived

**Figure 1:**
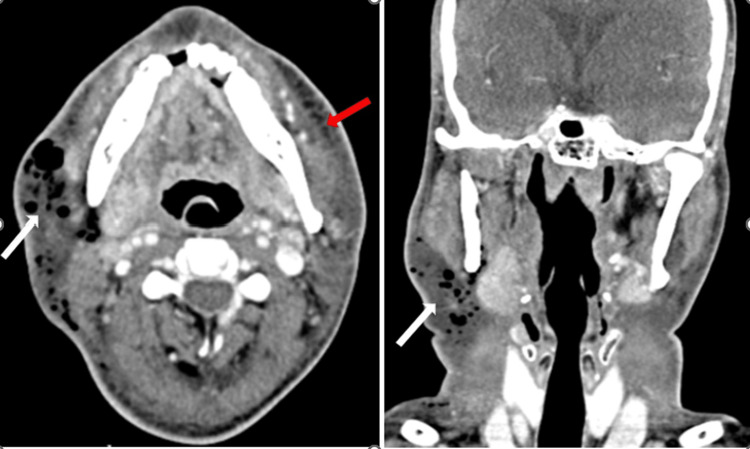
(Case 1) Contrast-enhanced CT of the neck (axial and coronal) shows an extensive ill-defined fluid collection (white arrows) containing multiple air pockets involving the subcutaneous plane of the neck bilaterally and extending into the right submandibular space. Note the absence of a peripheral rim around the fluid collection. Significant soft tissue edema and stranding is also seen (red arrow).

**Figure 2:**
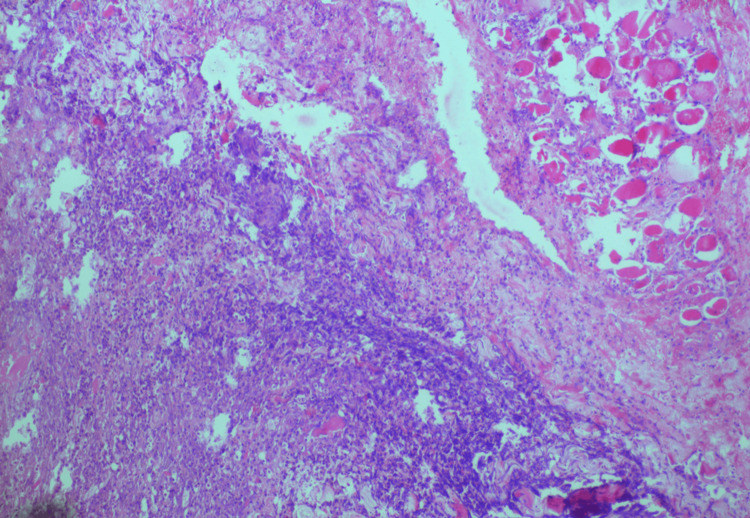
Histopathology image of case 1: necrosis of the fibromuscular and fibrocollagenous tissue

**Figure 3:**
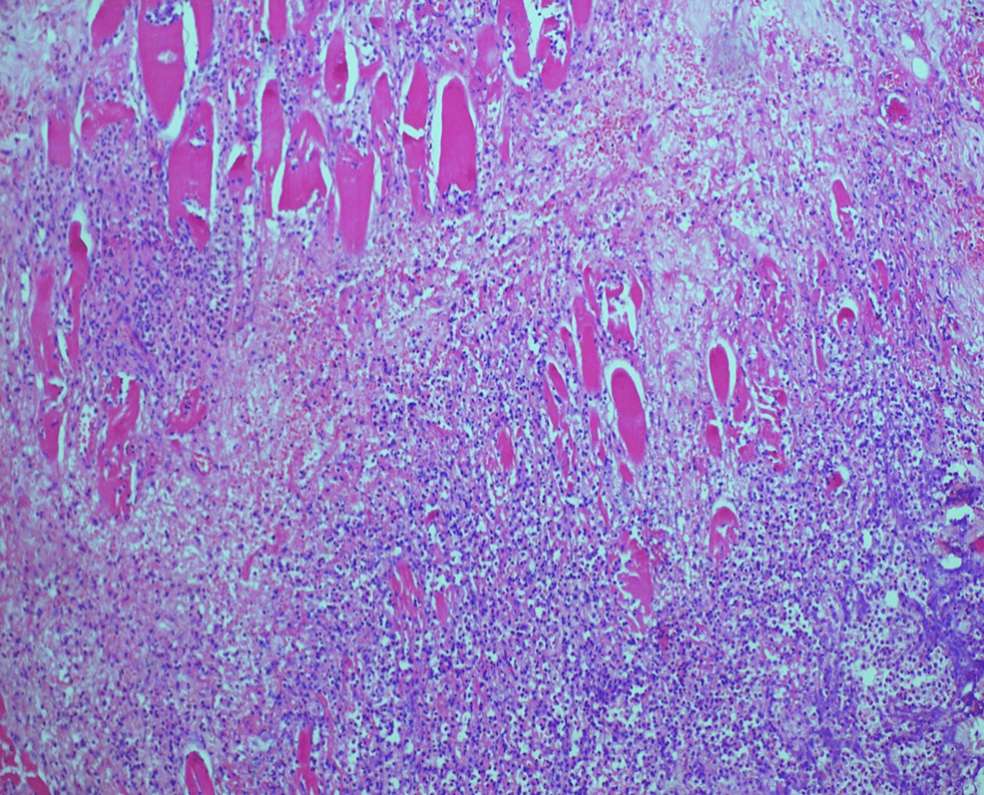
Histopathology image of case 2: hematoxylin and eosin (H&E) section shows necrosis involving the muscular and fibroconnective tissue along with a karyorectic debris

**Figure 4:**
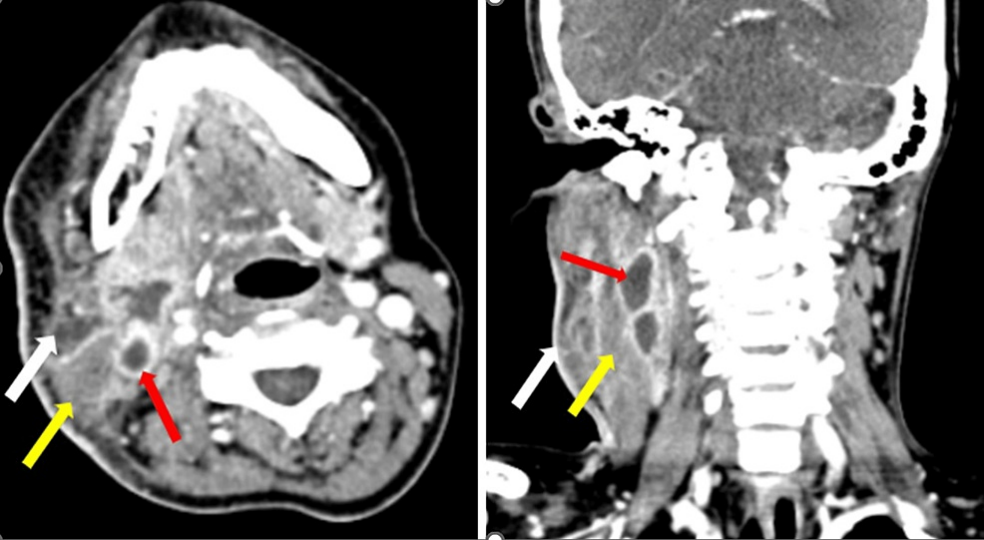
(Case 3) Axial and coronal contrast-enhanced CT of the neck reveals a bizarre-shaped hypodense fluid collection involving the superficial and deep facial planes of the neck on right side. The fluid collection shows a predominantly absent peripheral rim of enhancement (white arrows). However, a thick rind of enhancement is seen surrounding few fluid pockets suggesting abscess formation (red arrows). The right sternocleidomastoid muscle belly appears bulky and shows reduced enhancement as compared to the opposite side, suggesting myositis (yellow arrows).

**Figure 5:**
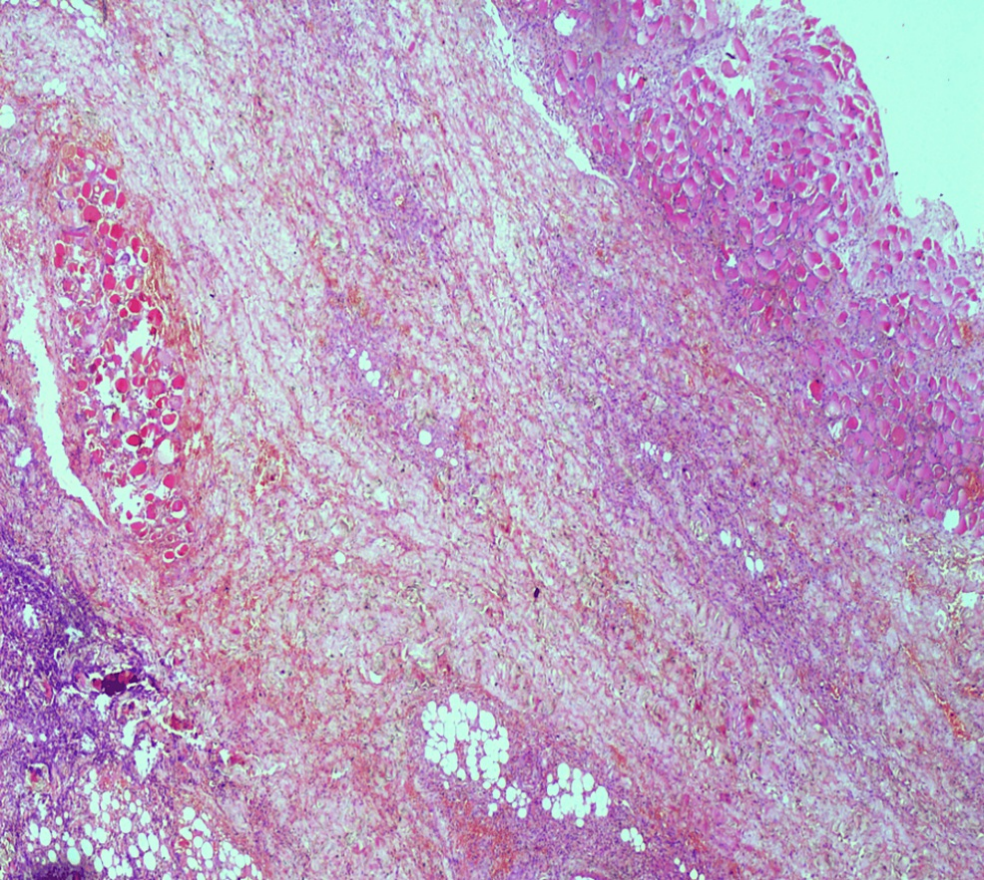
Histopathology image of case 3: hematoxylin and eosin (H&E) section shows necrosis involving the muscular and fibroconnective tissue along with a karyorectic debris.

**Figure 6:**
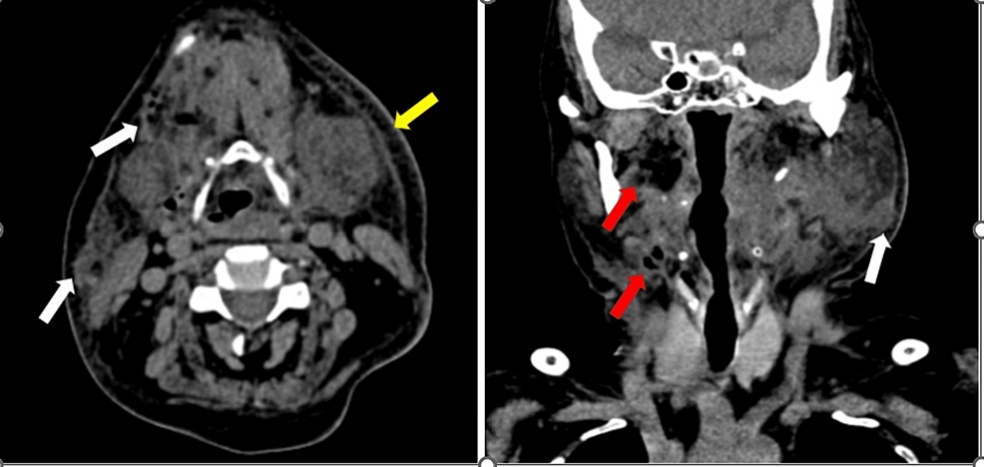
(Case 4) Plain CT neck (axial and coronal section) reveals an ill-defined hypodense fluid collection with multiple air foci involving the bilateral submandibular space platysma (white arrows), floor of mouth, and right parapharyngeal fat space (red arrows). Subcutaneous edema and stranding is also noted (yellow arrow).

**Figure 7:**
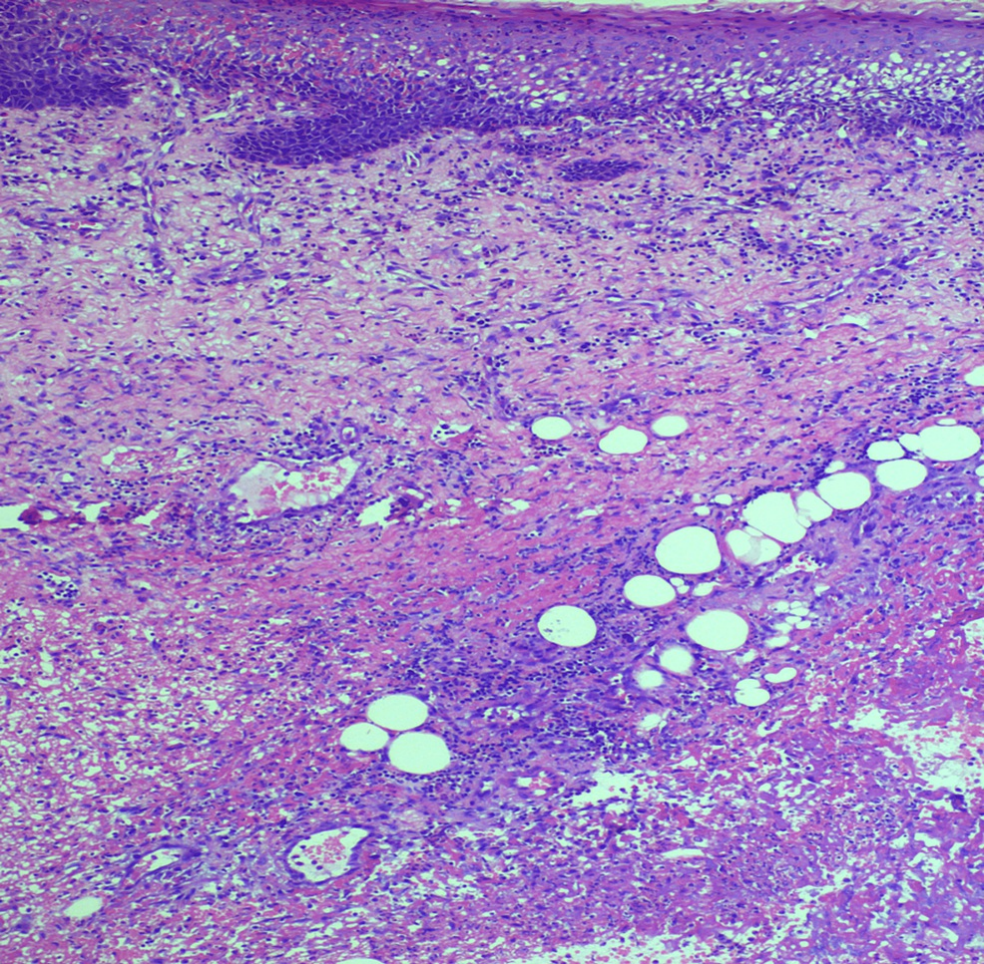
Histopathology report of case 4: necrosis of the fibromuscular and adipose tissue with an overlying stratified squamous epithelium.

## Discussion

The first description of modern necrotizing fasciitis was made by Joseph Jones, a military surgeon of the Army of America, but it was Wilson who coined the term necrotizing fasciitis [4].

Necrotizing fasciitis is a rare fulminant progressive soft tissue infection, with a high mortality rate if not managed timely and aggressively. It begins in the subcutaneous tissue of the superficial fascial plane and then involves the deep fascial planes with microvascular occlusion. This causes the superficial nerves to be damaged causing anesthesia [4].

The infection usually involves the second and third molars as their roots extend below the insertion of the mylohyoid muscle, which facilitates the spread of infection into the spaces. In deep neck space infections, there is skin redness, swelling, and induration with pus, whereas in necrotizing fasciitis, there is skin discoloration. In three cases, the submandibular and submental spaces were involved; in one case (case 1), there was a spread to the upper chest.

Uncontrolled diabetes remains the most common predisposing factor for compromising the immunity in most cases [2,4], causing longer hospital stays and more complications. This was similar to the findings of Zheng et al. who concluded that diabetics have a longer duration of hospital stay [5]. Among the two cases with diabetes, one (case 1) had a long-standing history of uncontrolled diabetes with irregular treatment and follow-up and improper diet control; hence, ancillary procedure, i.e., split skin grafting, had to be deferred due to raised blood sugar levels. The other patient was newly diagnosed with diabetes (case 4). Mostly, necrotizing fasciitis is a polymicrobial infection, but sometimes, the culture can be sterile as in the three cases.

The collagenase and hyaluronidase produced by the *Streptococcus *bacteria cause liquefactive necrosis of fat and fascia, and other bacteria can also be seen, such as *Staphylococcus* and *Prevotella*. This results in skin separation from the subcutaneous tissue with the production of dishwater pus pathognomic of it. If the infection still flourishes, then venous thrombosis and inflammatory cell infilterate spread to deeper fascial planes with vascular compromise, causing gangrene and skin necrosis [6].

The most common presenting symptom was tender neck swelling with skin discoloration in all cases. The increase in the glucose level is because of gluconeogenesis from the protein, resulting in hypoproteinaemia, which correlates with all our cases. Hyperglycemia impairs the function of leukocytes, causing less circulating lymphocytes and T-cells; hence, the body is unable to respond to infections [7]. Thus, blood sugar monitoring with strict glycemic control should be achieved for a good prognosis.

Inflammatory markers, such as C-reactive protein, rise with infection, necrosis, inflammation, and thrombosis. All the four cases had raised marker levels. The Laboratory Risk Indicator for Necrotizing Fasciitis score (LRINEC) score consists of the CRP, total WBC, hemoglobin, serum sodium, creatinine, and glucose value. A value less than five carries low risk, and more than eight carries a high risk of necrotizing fasciitis. A CRP value greater than 150 is highly suspicious of necrotizing fasciitis. As the inflammation becomes severe, the white cell count rises, marker CRP rises, hemoglobin levels drop, and albumin falls, which were seen in all the cases.

The factors that can affect prognosis include advanced age, anemia, hyperglycemia, involvement of multiple spaces, comorbidities, and presence of complications [8].

Radiological investigations should be done to assess the extent of spread of infection and the spaces involved. The case with uncontrolled diabetes (case 1) presented with breathing difficulty, dysphagia, and marked neck swelling extending to the upper chest with few days' duration along with skin discoloration and necrosis, so the case was taken for urgent neck exploration after doing radiology. The other cases also had unilateral neck swelling with restricted mouth opening and odynophagia. Ultrasonography of the neck was done in emergency basis in one of the case (case 2), and there was suspicion of an abscess. Hence, it was taken for incision and drainage, but necrosis of the tissue was seen intraoperatively, which was sent for histopathology and culture sensitivity. In the other cases, emergency contrast-enhanced CT scan of the neck with neck exploration was also done due to skin discoloration and pus discharge.

As all the cases were dealt with on an emergency surgical basis and managed with triple broad-spectrum antibiotics and regular aseptic dressing twice daily, none of the cases had complications and all survived, but one case required split skin grafting. Later, as per the culture sensitivity report, they were shifted to culture-directed antibiotics for seven to 10 days in the case of non-diabetics and for 10 days or more in the cases of diabetics. Oral antibiotics were also given for seven days on discharge. It should be differentiated from deep neck space infection, where there is skin redness, swelling, and induration with pus, whereas in necrotizing fasciitis, there is skin discoloration, bullae, pus along with slough, and necrosis.

Hence, our aim should be to promptly diagnose the cases, with aggressive surgical intervention and regular and frequent debridement to improve drug bioavailability in devitalized tissues. This helps to drain the loculated collection in fascial planes until viable tissues are encountered. It also limits the spread of the infection to the mediastinum via the retropharyngeal (danger space) or prevertebral, carotid spaces. This prevents complications, such as mediastinitis, pleural effusion, airway obstruction, respiratory compromise, and rupture/thrombosis of major blood vessels.

## Conclusions

Cervical necrotizing fasciitis is difficult to diagnose, but a high index of suspicion is needed in a rapidly progressive, fulminant life-threatening disease of the soft tissue and fascial planes. The diagnosis can be made simpler with a detailed clinical examination, radiology, and laboratory indices. If there is airway compromise, then securing it should be the priority with early and aggressive surgical debridement, Culture-directed broad-spectrum antibiotics should be started along with nutritional and hemodynamic support to improve the prognosis and reduce complications.
